# Spreading dynamics in a cattle trade network: Size, speed, typical profile and consequences on epidemic control strategies

**DOI:** 10.1371/journal.pone.0217972

**Published:** 2019-06-10

**Authors:** Aurore Payen, Lionel Tabourier, Matthieu Latapy

**Affiliations:** 1 LIP6, UMR 7606, Sorbonne Université, CNRS, Paris, France; 2 AgroParisTech, Paris, France; INSERM, FRANCE

## Abstract

Infections can spread among livestock notably because infected animals can be brought to uncontaminated holdings, therefore exposing a new group of susceptible animals to the disease. As a consequence, the structure and dynamics of animal trade networks is a major focus of interest to control zoonosis. We investigate the impact of the chronology of animal trades on the dynamics of the process. Precisely, in the context of a basic SI model spreading, we measure on the French database of bovine transfers to what extent a snapshot-based analysis of the cattle trade networks overestimates the epidemic risks. We bring into light that an analysis taking into account the chronology of interactions would give a much more accurate assessment of both the size and speed of the process. For this purpose, we model data as a temporal network that we analyze using the link stream formalism in order to mix structural and temporal aspects. We also show that in this dataset, a basic SI spreading comes down in most cases to a simple two-phases scenario: a waiting period, with few contacts and low activity, followed by a linear growth of the number of infected holdings. Using this portrait of the spreading process, we identify efficient strategies to control a potential outbreak, based on the identification of specific elements of the link stream which have a higher probability to be involved in a spreading process.

## Introduction

Production of dairy and meat products is a major economic field in Europe. Fighting disease spreading is thus a key issue for the protection of economic interests, as well as human health and animal welfare. Among the various routes to infect holdings, such as contamination by wildlife or contacts between herds in pastures, cattle trade movements spread pathogens at national and international levels, and are thus a major way of infection. People and decision makers in Europe have recently become more aware of the problem. In particular, since the Bovine Spongiform Encephalopathy crisis of 1996, each state of the European Union has to identify every bovine on its territory and to register cattle trade movements. The *Base de Données Nationale d’Identification* (BDNI) database, which is the focus of this work, is the French enforcement of this decision. This kind of data is characterized by the availability of temporal information through the record of dated cattle exchanges. The aim of this study is to fully capture the importance of temporal information when modeling disease spreading in order to evaluate potential outbreak sizes and to be able to characterize spreading speeds.

### State of the art

Modeling disease spreading has been a hot topic for years [1–5]. Essentially, this issue relies on two ingredients: the infection model and the structure of underlying contacts. Concerning the infection model, the simplest one in the literature is certainly the SI model, which considers that nodes are in one of two states (or compartments): susceptible or infected. Susceptible nodes become infected when interacting with other infected nodes, according to a given probability. Many other compartments can be added, depending on the specific disease under study to make this propagation model more realistic [2]. Among others, the SIR model adds a Recovered state, which simulates immunity after having recovered from a disease; or the SIS model, where nodes return to a Susceptible state after being infected with a certain probability per time step or after a fixed time. For all these models, the system eventually reaches a stationary state which reveals the final fraction of the population being infected according to the chosen parameters: infection and recovery rates, delay before immunity, etc. (see [6] for example).

The full definition of the problem demands to describe how infected agents encounter susceptible ones. A basic hypothesis is to suppose that the nodes are able to interact with all the population with the same probability. Such a population is said to be *fully mixed*. However, real populations are known to present different encounter behaviors depending on individuals. Since the late 1990s, complex networks analysis has brought a new momentum to the domain by emphasizing the role of this second ingredient, i.e. the structure of the contacts underlying spreading phenomena [7]. Using a simple and general graph-based representation, it allows to represent a variety of situations and scales. For instance, the nodes of a graph may be individuals [8], locations such as cities [4] or agricultural holdings—which are our objects of interest. The versatility of these models also allows to represent other kinds of spreading phenomena, which characteristics are supposed to be resemblant to infection spreading, such as a rumor spreading in a social network [9]. Taking into account the characteristics of the network has led to identify issues which result from its heterogeneities. For example it has been shown that epidemics tend to remain endemic on a heterogeneous structure [7]. Also, it has been stressed the prominent roles of specific nodes [10], and adapted epidemic control strategies ensued [11, 12].

In parallel of the studies emphasizing the role of heterogeneous contact patterns on the spreading dynamics, other works have brought into light the fact that temporality of interactions also plays an essential role. Indeed, in many real world situations, the number of interactions per node varies over time, and nodes that were interacting a lot during a period can suddenly turn inactive. For example, the seminal work of Morris *et al*. on HIV [1] showed that concurrent partnerships could considerably increase the impact of spreading compared to sequential contacts. As time-labeled data was not easily available, the body of literature on this aspect grew mostly in the last ten years. Moreover, early attempts at using temporal information tend to use snapshot-based description of the data [13, 14]. It consists in representing the data during a given period as a graph, where a link between two nodes represent the existence of at least one interaction between these nodes during the period considered. Thus, such representation allows to use the toolbox provided by graph theory. However, choosing appropriate time windows to analyze temporal data is known to be a difficult question [15], even impossible to manage when several timescales are intertwined. It has been suggested quite early that burstiness and consequently the heavy-tail of the inter-event times distribution play a critical role in the spreading process [16–18]. Other works have underlined that the bulk of this distribution cannot be neglected and have laid stress on the relative order of the events [19]. More generally, the dynamics of links considered separately cannot account alone for the global spreading process, and several studies [20–23] evaluate with null models the impact of the correlation between the structure and the dynamics of the network.

The study of both the static and dynamic properties of the contact networks are relevant in the context of animal trade networks. Indeed, we have already mentioned that knowing the structure of the network is essential to identify efficient epidemic control strategies [3]. Consequently, many studies have focused on the description of such network data, sometimes limited to a static description [24]. When temporal information is included, it is often scrutinized using graph snapshots, but recent works tend to consider snapshots as sequences of graphs, and analyze spreading processes that span over several snapshots [5, 25–32]. While there is still an important focus on graph features and representations, these studies also show an increasing interest to account for the intrinsically dynamic nature of the data. Some of these works examine purely temporal characteristics and the stability of the features observed through time [27, 29, 30]. Others introduce or use concepts such as infection chains or disease flow centralities, which allow to investigate the limits of purely graph-based representations and discuss the potential size of an epidemic [25, 26, 32] as well as epidemic risk in general and ways to mitigate it [5, 28–30]. We will come back to these points in more details later.

### Position and contribution

Our study focuses on the French cattle trade network from 2005 to 2015, as it appears in the BDNI. Other works have described previous versions of this database: [24] described year 2005, and [30] described the 5-years period from 2005 to 2009. Here, we do not aim at modeling the propagation of a specific disease, with a precise compartment model or a very fine-grained description of the agents of the system—in the spirit of [33] for example. We rather consider this study in the line of [34]: we explore the structure of the dataset using a simple epidemic model, which is a deterministic SI model, with an infection probability during a contact set to 1. This model can be understood as a proxy to represent a worst case scenario. Besides that, many conclusions on spreading characteristics remain valid for more complex propagation models in spite of its simplicity. For example, if we are looking for potential super-spreaders of an infection, there is a high probability that a node identified as such with a SI model would also be a super-spreader using a more elaborate model. [35] also describes the SI model as a good mean to explore the first stages of an infection spreading. From our point of view, a decisive argument to use such a model is that one may consider that it is rather a measurement probe of the structure of the data, as it is deterministic.

We seek properties of the temporal structure of the dataset which have an important impact on spreading processes in the specific case of this cattle trade dataset. Indeed, cattle trade networks exhibit specificities that we aim at pointing out, and in that sense our work is close to studies such as [30, 32].

The contributions of this work are the following:

- We evaluate on the BDNI the impact of the events chronology on the reachability of nodes. These measurements are close or similar to what has been observed in previous studies on the German pig trade network [29, 32]. On this point, we confirm their observations and suggest that most animal trade networks would probably lead to the same qualitative conclusions.
- We propose a new measurement of the spreading impact which allows to differentiate spreading scenarios by also accounting for the spreading speed in a SI context.
- Then, we propose a simple and intuitive model to describe a spreading phenomenon, which allows to describe the process with few parameters. This model corresponds to a specific kind of scenario which seems to be the norm in the BDNI with the spreading model that we have chosen.
- Finally, based on the former observations and definitions, we propose suited target control protocols. In particular, we propose to use as a cost unit the actual atom of information in a link stream, which is an interaction. We will see that it deeply affects the intuition that a user can have of the efficiency of a target control strategy. And consequently, we bring into light that link-based strategies, which directly use the temporal nature of the data are more efficient than usual node-based strategies in the literature.

In order to reach these goals, we first present some characteristics of the dataset, focusing on properties which have a direct impact on the spreading dynamics examined later. Then, we discuss the infection model at the heart of this work and the sequences of infections that ensue in a static and a dynamic context, comparing the number of reachable nodes in both cases. We propose a simple model of the sequences of infections based on a two-phases description of the process, which provides a schematic description of the dominating spreading scenario. Finally, we propose to rethink standard targeted control strategies in the light of the observations made, in particular we propose strategies based on the descriptions of the sequences of infection which prove to be efficient in the context of the BDNI.

## Materials and methods

Throughout this work, we deal with the BDNI, which records bovine trade movements in France. We have access to eleven years of data from 2005 to 2015. The access to the BDNI is not public, but can be obtained through a specific agreement with the French ministry of agriculture. It contains approximately 148 million animal transfers. Animals are often traded in batches, and as we do not model an infection at the animal level, we focus on batch transfers between holdings, which are dated with a daily granularity. Batch sizes are not investigated in this work. Moreover, different types of holdings feature the data: farms, markets, assembly centers, slaughterhouses and knackery premises. Movements to slaughterhouses and knackeries are dead-ends concerning disease propagation, therefore, we exclude them from our data. After this basic preprocessing, there are around 32,600,000 time-labeled batch movements in the dataset. Among the remaining 300.000 holdings, there are 90 markets (0.03%), about 2800 assembly centers (0.9%), and the rest are farms.

### Data modeling

One needs a formalism to represent time-labeled data that contains the information of the interactions which can support the infection spreading. Such data is often referred to as a temporal network, which is a general term to refer notably to a collection of triplets *(t, i, j)* meaning that node *i* interacts with *j* at time *t* [36]. Depending on the data specifics (for example interaction durations), authors adapt the formal tools to the context and their needs. Recently, specific formalisms have been developed to define mathematical tools and algorithms adapted to these data. In this study, we use the link stream formalism [37], which favors an intuition based on the idea that such data are best represented by taking into account both its graph-related and time-series related characteristics. Other formalisms exist with similar benefits, like for instance TVG [38], but we use here the link stream formalism that emphasizes the streaming nature of data. It also allows to describe the temporal network with continuous time, though we do not use this property of the formalism in this work. In a few words, a link stream L is a set of nodes *V*, a set of temporal links or interactions *E*, and a set of interaction times *T*. An interaction is a triplet (*t*, *i*, *j*) ∈ *E*, with *t* ∈ *T* and (*i*, *j*) ∈ *V* × *V*.

Here, a directed edge from holding *i* to holding *j* on day *t* corresponds to a triplet *(t, i, j)* in the data. In most experiments conducted in this work, we focus on temporal parts of the dataset. We mainly use year 2015, and check that the results are consistent for other time periods.

### Implementation

Throughout this work, our computer implementations have been achieved in python. Specific graph-based computations (such as betweenness centrality) are realized using the NetworkX library (https://networkx.github.io/). To give the reader an idea of the order of magnitude of the computational times, a typical batch of 1000 spreading runs (such as the ones described later in the paper, with Δ = 4 weeks) takes approximately 1 hour. Consequently, it may be useful to use a lower level, more efficient programming language in order to scale up the simulation parameters.

### Characteristics of the BDNI

In the rest of this section, we report a few features of this dataset, which have a direct impact on the spreading dynamics, as we will see later on.

#### Temporal variations of active nodes

**Fig 1:**
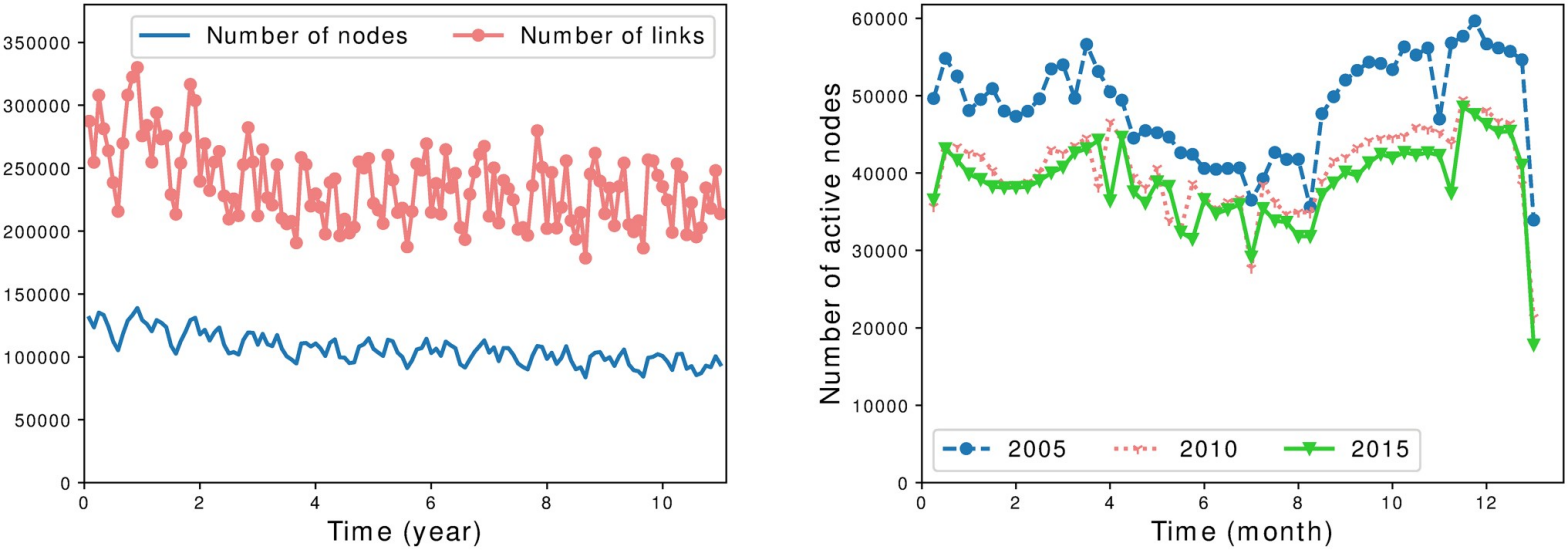
Number of active nodes. Left: number of active nodes and number of transfers per month from 2005 to 2015. Right: number of active nodes per week over several years. A node is active if it is involved in at least one transfer during the period considered.

A holding is said to be active during a given period if it sent or received at least one batch during this period. We show in Fig 1 the number of active holdings (or nodes) and the number of batches exchanged over several aggregation periods. Of course, only active nodes can relay an infection, so it gives insight on how important is the risk of spreading a disease in case of an outbreak depending on the period considered.

We can see that the number of active holdings as well as the number of transfers is fluctuating: the overall trend is a decrease over the years. It is consistent with the observations on years 2005 to 2009 in [30], and it is probably a consequence of the holding merging phenomenon in France. We also see periodic activity patterns which are visible when measuring the number of active holdings per week. Overall, there is a higher activity level during early spring and autumn, and a lower level during summer and at the end of the year, which is consistent with the common knowledge of the field. Note that at a daily scale (not represented here), we observe peaks of activity on Mondays, then the activity decreases throughout the week and is at its lowest level during weekends.

#### Heterogeneity of nodes degree and activity

We now investigate the number of neighbors of a node, that is to say the number of holdings from and to which it receives and send batches. As previously discussed, the heterogeneous structure of the network is known to have a significant impact on spreading processes.

**Fig 2:**
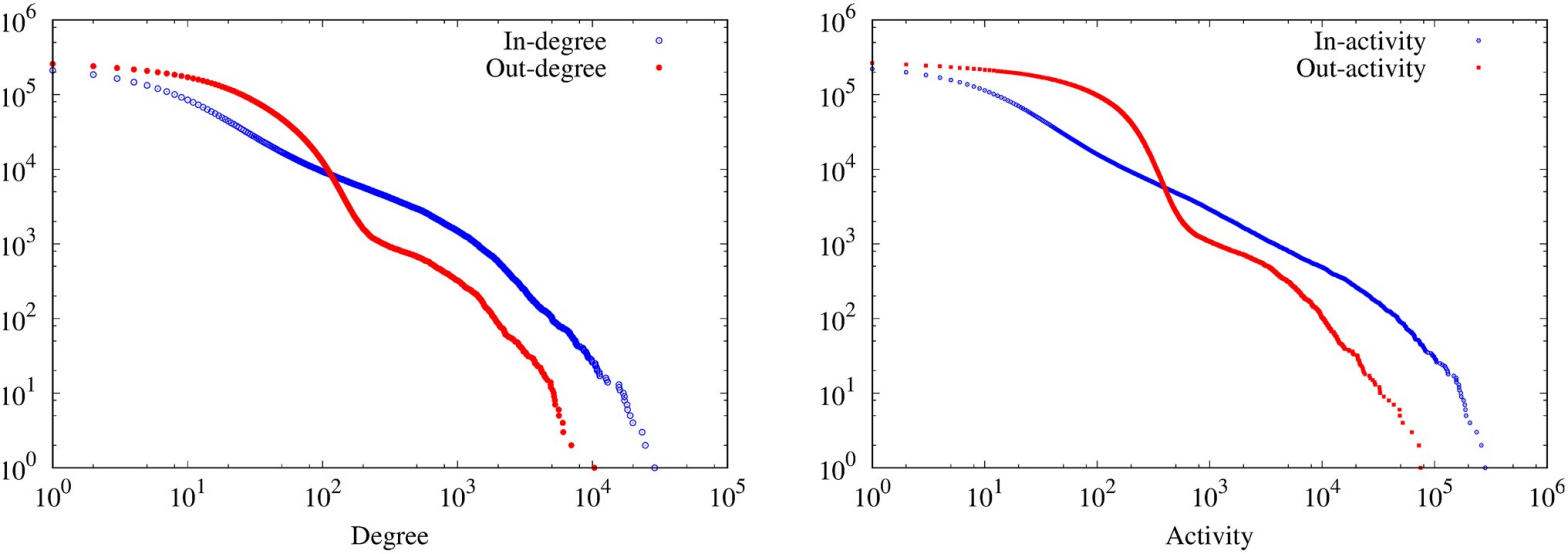
Degree and activity inverse cumulative distributions. Inverse cumulative degree (left) and activity (right) distributions over 11 years of data collection. Distributions are clearly heterogeneous, but while in-degree and in-activity ICDs are relatively close to power laws, out-degree and out-activity exhibit two modes, with sudden drops at intermediary values.

We measure the inverse cumulative distributions (ICD) of in and out degree of the nodes, as well as the inverse cumulative distributions of in and out activity, that is to say the number of in and out transfers a node is involved in. We plot the results obtained for the year 2015 in Fig 2. Similar results are obtained for every year of the BDNI. The degree distributions are heterogeneous, which is also consistent with the literature of the field [27, 30, 32]. Note that the in and out-activity ICD have different shapes as the in-activity ICD is closer to a power-law distribution; by contrast, the out-activity ICD exhibits much fewer high activity nodes and more nodes with low and intermediary values, with a significant drop when the activity reaches approximately 100 transfers.

A few nodes are in or out-hubs. For example on the 2015 data only, if we arbitrarily set the threshold to 100 batches bought or sold, around 1.7% of nodes with in-degree > 0 are in-hubs, while 0.3% of nodes with out-degree > 0 are out-hubs. Markets and assembly centers are overrepresented among hubs, as they account respectively for about 2.7% and 39.1% of in-hubs and 7.2% and 91.7% of out-hubs. They also have a much larger average out-degree (markets: 263, centers: 105, farms: 5 in 2015) and average in-degree (markets: 721, centers: 292, farms: 3 in 2015).

#### Asymmetry of interactions

It was observed that the French cattle trade network is asymmetric, in the sense that the existence of a link from i to j does not involve in general the existence of a link from j to i [30]. An indication of this property can be seen by comparing the number of holdings buying and selling cattle. For instance in 2015, 95% of active holdings are selling animals, while only 58% are introducing new animals in their herds. This property is also measured using the reciprocity ratio *RR*, which is defined as the fraction of reciprocated links during a given period:

$$\begin{array}{c} RR=\frac{\text{number}\;\text{of}\;\text{reciprocated}\;\text{links}}{\text{total}\;\text{number}\;\text{of}\;\text{links}} \end{array}$$

We measure that this ratio is indeed between 0.10 and 0.13 for yearly networks, and between 0.06 and 0.09 for monthly networks. Note that the *RR* is mostly relevant when compared to a reference model, as underlined in [39]. Here, it is hard to decide what is an appropriate reference model as a holding in the network can only reach a limited number of other holdings, due for example to constraints related to geography or to the production chain. However, a layperson could think that if an animal can be sent from holding *A* to holding *B*, then the reciprocal is true and therefore would expect a rather high probability of observing this situation. But these measurements indicate that this is actually a rare situation and thus, the directedness of traffic flows should not be neglected, as it certainly impacts potential infection propagations.

We have reported a few characteristics of the dataset which have a direct impact on spreading simulations. A more detailed description of the BDNI is not the topic of this work, however we report that the dataset exhibits characteristics which are consistent with measurements made on previous analyses of the BDNI [24, 30]. Moreover, other bovine trade networks exhibit similar structural properties, for example the dataset has qualitatively similar distributions of inactivity periods as the one observed in the Italian cattle trade network [27]. Even other animal trade networks have common traits with the one that we have used, such as pig trade in Germany [32], which suggests that results presented in the following might be general to animal trade networks.

## Results

### Node reachability in snapshot-based vs temporal representation

A usual question when considering the spreading of an infection on a network is which nodes can be reached by the infection, knowing already infected nodes. The point investigated in this section is the closely related issue of evaluating the maximum number of nodes that can be reached in the worst case scenario where any transfer from an infected holding to a susceptible one infects it systematically. In particular, we compare the results obtained when the data is described with static graph snapshots to the case where the data is described as a truly temporal network.

#### Reachability in a directed network

**Fig 3:**
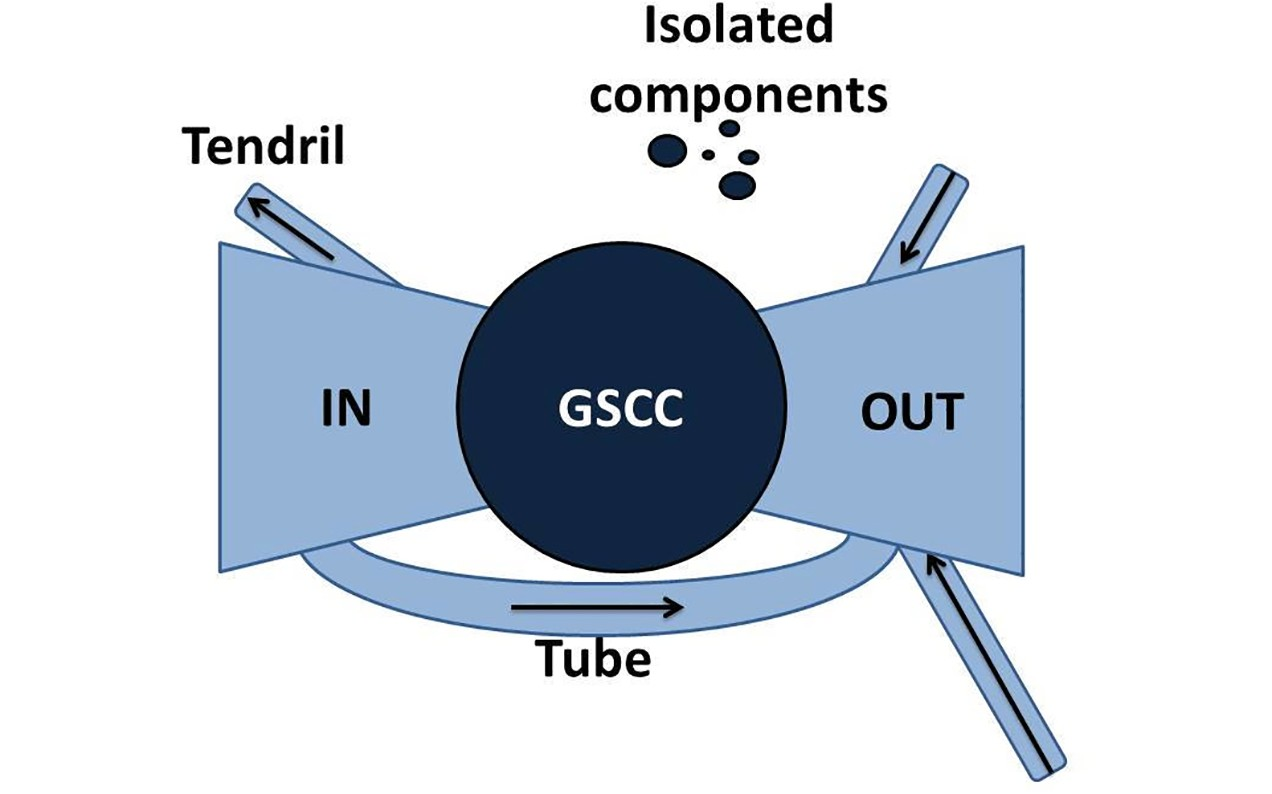
Bow-tie structure of a directed graph. The bow-tie map of a directed graph exhibits the different parts of any directed graph with a giant weakly connected component: its GSCC, the relative in and out-components, tendrils and tubes. Adapted from [40].

We call snapshot of the data a static network representation which aggregates the interaction data during a given time window. Using this representation, the spreading process is considered to take place on the network over the aggregation period only, and is therefore equivalent to a spreading process on a graph. In this case, the question of the reachability of nodes can be understood through a large-scale description of the directed network. Indeed, real-world directed networks often exhibit bow-tie structure, as represented in Fig 3, which was originally used as a large-scale map of the world wide web [40]. A giant weakly connected component gathers most nodes of the network, and such component can be divided in the following way:

- a central bulk which is a giant strongly connected component (GSCC), where any node can reach any other node following a directed path;
- a component upstream to the GSCC, called in-component, where nodes can reach nodes of the GSCC following a directed path, but cannot be reached by the nodes of the GSCC;
- reciprocally, some nodes are downstream to the GSCC: they can be reached from the GSCC following a directed path, but cannot reach it, they constitute the out-component;
- the remaining nodes of the giant component are part of structures called tendrils (going out from the in-component without reaching the GSCC, or going in the out-component without coming from the GSCC) and tubes (connect directly the in- to the out-component, without going through the GSCC).

Finally, other nodes in the network are in small weakly connected components.

Snapshots of the BDNI exhibit a structure of this kind. For example, the directed graph obtained from the interactions of year 2015 has 176.771 active holdings that year, among which 98% are in the giant weakly connected component, 42% are in the GSCC, 47% in its in-component, 3% in its out-component, 6% in tubes and tendrils. These percentages are stable from one year to another. Note that the aggregated graph at other timescales (monthly, quarterly) also exhibit a bow-tie structure.

We consider a deterministic SI model on such a structure, starting from a random node in the network (the seed), and investigate the number of nodes reached at the end of the process. The result can be easily anticipated from the bow-tie: if the seed is chosen in the GSCC, the infection reaches all nodes located in the GSCC and all nodes in the out-component; if it is located in the in-component, it reaches nodes of the GSCC and of the out-component and the nodes of the in-component located on a directed path from the seed to the GSCC, etc.

#### Spreading cascades and reachability in a link stream

**Fig 4:**
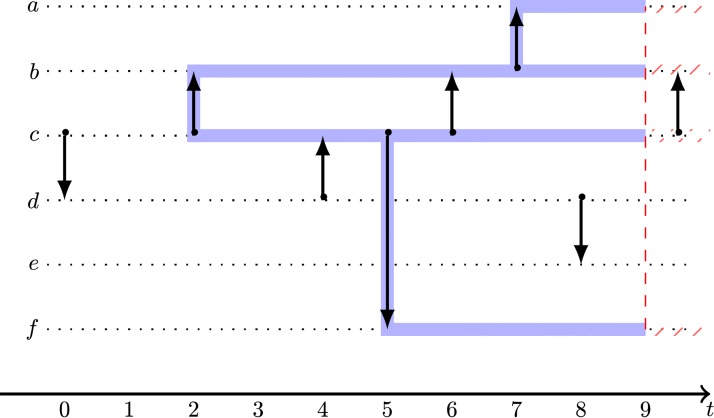
Spreading cascade. Representation of a spreading cascade (in blue) starting from node *c* at time *t*_0_ = 2, with overall time duration Δ = 7. The spreading stops at time *t*_0_ + Δ = 9 (in red). Arrows represent directed interactions from node *i* to node *j* at time *t*, they are supposed to be instantaneous.

In the context of temporal networks, we define what we call a *spreading cascade* in the following of this work. We simulate a deterministic SI spreading model starting from a seed node at a given time *t*_0_, with an infection active only during a predefined period of time Δ. By contrast with the snapshot case, the spreading follows the chronological order of the interactions. The spreading cascade is the link stream that is built from this propagation process. Precisely, when there is a directed interaction (*t*, *i*, *j*) from an infected node *i* to a susceptible one happening between *t*_0_ and *t*_0_ + Δ, then *j* is infected, and it is included as a node of the cascade and the triplet (*t*, *i*, *j*) is added to the temporal links of the stream. An example of such cascade is represented in Fig 4. The number of nodes in the cascade is thus the number of reachable nodes. A similar measurement can be found in the epidemiology literature, it has been defined and used under the name *out-component respecting temporal sequence of contacts* [29], and a previous version of it with no time limit Δ is called *outgoing infection chain* [5, 25, 41, 42].

#### Comparing maximum outbreak sizes

We now compare experimentally the potential sizes of the outbreaks according to the SI model with the snapshot-based representation and with the link stream representation using the measurements aforementioned. First, we need to precise some details of the experimental setup. Suppose that we consider snapshot of duration *T*. To make the comparison as fair as possible, we compare the number of reachable nodes on these snapshots to spreading cascades of duration Δ = *T* in the link stream. In the rest of the article, we use three timescales which are typically used to investigate animal trade data: 4 weeks, 3 months and 1 year [26, 30]. We run a SI model using successively as a seed every active node during the period of interest. In the temporal network case, the starting time *t*_0_ of the cascade is decided as follows: we draw a random point in time, then *t*_0_ is chosen as the moment of the next outgoing interaction from this node (if it exists). Again, for the sake of clarity, we only present results obtained on year 2015 for Δ = 4 weeks and 3 months, but the results obtained on other years are consistent. Concerning Δ = 1 year, the starting points of temporal cascades are drawn during year 2014, as having starting points in 2015 would not allow to have 1 year long cascades.

**Fig 5:**
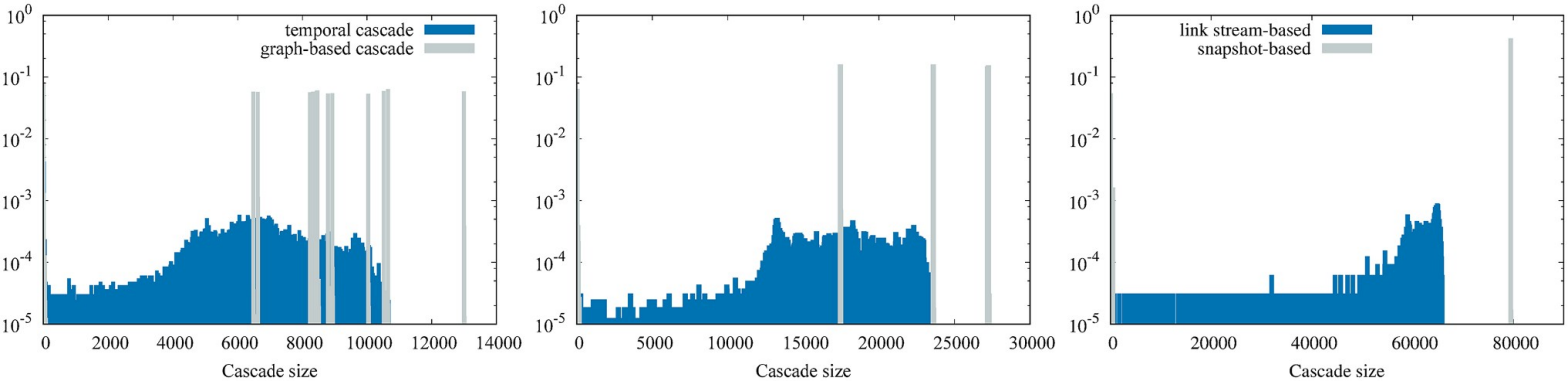
Outbreak sizes. Distributions of outbreak sizes using graph-based cascades (snapshot-based representation) and temporal cascades (link stream representation). Δ = 4 weeks and 3 months distributions are built from all the outbreaks computed during year 2015, while Δ = 1 year distribution results from the outbreak computed on years 2014 and 2015. While graph-based cascades are distributed in a few modes while temporal cascades reach less nodes and do not exhibit such modes.

In Fig 5, we represent the size distributions obtained using the three timescales investigated. Let us first analyze the yearly network: it clearly displays two different situations: most of the outbreaks are large (92% reaching more than 79,000 nodes), and the remaining ones are small (between 1 and 500 nodes). In other words, the distribution exhibits two modes. This is a direct consequence of the bow-tie structure mentioned above: if the seed is in the GSCC or the in-component, it will reach all nodes downstream, that is to say the GSCC, the out-component and possibly a few nodes of the in-component (up to 79,510 nodes, that’s 45.0% of active nodes). Qualitatively the results are quite similar at smaller timescales in the sense that we observe several modes. In fact, static distributions are obtained by putting together outbreaks over each 4 weeks and 3 months snapshot in 2015, but if we consider each of these snapshots separately, it also has only two modes. Besides that, the largest outbreaks reach a smaller fraction of the nodes active that year (7.5% on 4 weeks networks, 15.5% on quarterly networks). This observation confirms the fact that yearly animal trade mobility is not at all the repetition of movements occurring at a monthly or quarterly scale, which is consistent with previous studies [26, 27, 30].

The results for propagation cascades using a link stream model show that maximal outbreak sizes reach less nodes than their static equivalent: 6.0% for 4 weeks cascades of all active nodes in 2015 (to be compared to 7.5%), 13.2% for quarterly cascades (comp. to 15.5%), 39.3% for yearly cascades (comp. to 45.0%). Consequently in a snapshot-based approach, the maximum outbreak size is overestimated by a factor 1.25 in the case of 4-weeks experiments (1.17 and 1.15 for quarterly and yearly timescales). Note that this overestimation factor is also measured in [32] and named *causal error*, as it represents the error committed when neglecting the causality imposed by chronological order. It shows that neglecting temporal information leads to significant overestimates of the possible size of epidemic outbreaks. We also observe in Fig 5 another striking fact: whereas yearly, quarterly and monthly snapshots consistently lead to different modes of outbreak sizes (a few small ones and a majority reaching a large part of the network, and none in-between), a temporal network representation leads to a continuum of cascades sizes, from small to large ones.

In short, taking into account the chronological order of the events reduces the systematic overestimation that a snapshot-based representation implies, and gives a more accurate view of the size distribution, which is continuous rather than discretized in a few modes.

Similar results have been shown in [29, 32] in the case of the German pig trade network. The authors observed that spreading cascades in this static directed graph are distributed in modes, by contrast with spreading cascades in a temporal network. They also observed that the maximum outbreak size is overestimated typically by a few tenths when using a snapshot description. Our work confirms the assumption made in [29] that this result is true for other animal trade networks, precisely the French bovine trade network. As the distribution of outbreak sizes in the snapshot case stems from the bow-tie structure of the directed network, we expect that it generalizes to many other contexts.

### Propagation profiles and modeling

The number of individuals reached by an epidemic-like process in a population can be schematically represented by a S-shape curve. First, there is often a slow initial growth, then it takes off and grows rapidly, in a way which is typically superlinear (e.g., [22]) and finally saturates when all reachable nodes are indeed reached. Of course, we expect that a SI spreading model behaves similarly on the BDNI dataset. However, this may correspond to very different spreading scenarios which depends on the duration of each of these schematic phases and on the timescale of observation.

#### Differentiating profiles with speed

**Fig 6:**
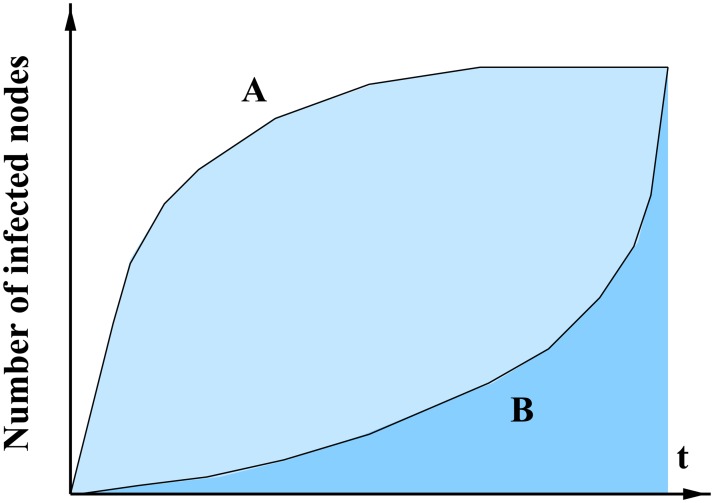
Spreading scenarios. Comparison between two schematic spreading scenarios. A is a *fast-then-saturating* scenario, which means than the infection spreads rapidly during the early days, and then the infection rate decreases. B is a *slow-then-expanding* scenario, which means than the infection spreads slowly at the beginning, and then the infection rate increases.

To discriminate between various spreading scenarios, such as the ones represented in Fig 6, the size of the outbreaks is not sufficient. One key advantage of the link stream modeling is that it allows to account for the speed of the spreading. In order to evaluate the speed of a cascade with a simple scalar measurement, we define the *Area Under the Infection Curve* (*AUIC*) as:

$$\begin{array}{c} AUIC\left(\Delta \right)=\int_{t_{0}}^{t_{0}+\Delta }n\left(t\right)dt \end{array}$$

where *n*(*t*) is the number of infected nodes at time *t*. Fast-then-saturating spreading scenario will have large *AUIC* while slow-then-expanding scenario will have low *AUIC*.

**Fig 7:**
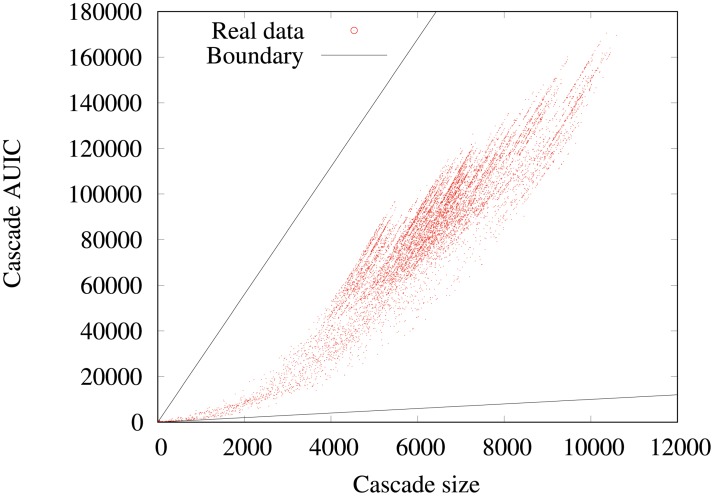
AUIC vs number of infected nodes (4-weeks): Real data. Scatter plot of the 4-weeks cascade sizes and *AUIC* in the real data. Each point is a cascade of the sample. The black solid lines represent the theoretical boundaries outside which there is no cascade, because of the theoretical relationship between the *AUIC* and the size of a cascade. The scatter plot exhibits a wing-like envelope and is structured as a beam of parabolic curves.

Now, we investigate on the data how the *AUIC* depends on the final number *n*_*f*_ = *n*(*t* = *t*_0_ + Δ) of infected nodes in a cascade. We plot in Fig 7 the corresponding scatter plot for the year 2015 data with Δ = 4 weeks. We observe that for a given number of infected nodes, the corresponding *AUIC* covers a broad range of values, especially for large cascades. For example for *n*_*f*_ = 6000, the *AUIC* ranges from 40, 000 to 100, 000. Yet, when comparing to the theoretical upper and lower bounds of cascades *AUIC* for a given *n*_*f*_, we observe that we only cover a small part of the possible spreading scenarios. These theoretical bounds correspond to infections with minimum and maximum possible *AUIC*. For a given final number of infected nodes, it corresponds to an infection where all nodes except the seed are infected on the last day (minimum) and to an infection where all nodes are infected on the first day (maximum). Moreover, the scatter plot has a very specific shape: it exhibits a wing-like envelope, and a closer examination indicates that it is a beam of parabolic curves passing by the origin of the plot.

**Fig 8:**
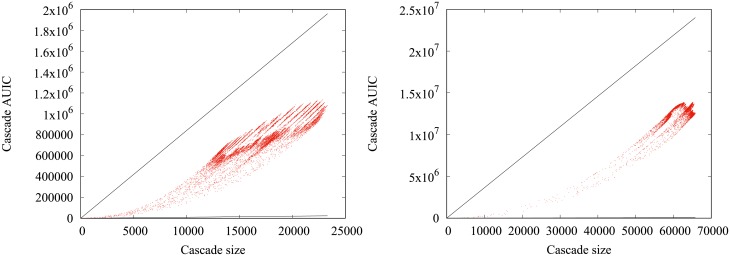
AUIC vs number of infected nodes (4-months, 1-year). Comparative scatter plots of the cascade sizes and *AUIC* in the real data for quarterly (left) and yearly (right) cascades. Each point is a cascade of the sample. The black solid lines represent the theoretical boundaries outside which there is no cascade.

When increasing Δ, the wing-like envelope tends to get thiner, with a higher density of cascades concentrated on a few parabolas and large *AUIC*, indicating that cascades are less diverse. Yet, qualitatively similar observations can be made with Δ = 3 month and 1 year, as can be seen in Fig 8.

#### Modeling the propagation

**Fig 9:**
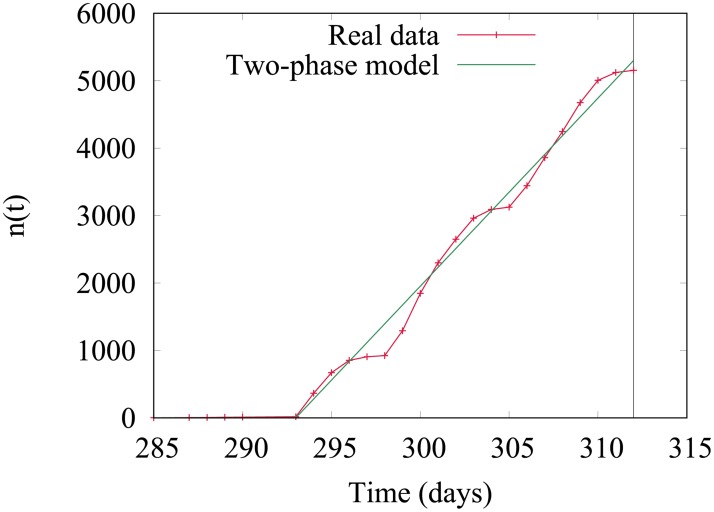
Fit of n(t). Example of the evolution of the number of infected nodes *n*(*t*) during a 4-weeks cascade and its fit according to the two-phase model, according to which the number of infected nodes first remains constant, and after a period *t*_*w*_ increases linearly.

In order to explain the scatter plot described in the previous section, we propose a simple model of the number of infected nodes as a function of time *n*(*t*). From the observation of large cascades, it seems that the most common spreading scenario is the following: during a first period that we call *waiting time*, very few nodes are infected, then the infection reaches a tipping point from which the number of infected nodes increases linearly. A typical fit of the evolution of the number of infected nodes as a function of time in a 4-weeks cascade is represented in Fig 9. During the growth phase, we can observe that the growth rate oscillates with a weekly period, which stems from the fact that cattle transfers are rare during the week-end.

In order to check whether this two phase process can account for the spreading characteristics, we fit its *n*(*t*) curve with a basic two-phase model $\hat{n}\left(t\right)$:

$$\begin{array}{c} \hat{n}\left(t\right)=\{\begin{array}{cc} 1 & \;\text{if}\;t\in [t_{0},t_{0}+w[\;\\ g\cdot (t-\left(t_{0}+w\right))+1 & \;\text{if}\;t\in [t_{0}+w,t_{0}+\Delta ]\; \end{array} \end{array}$$

The waiting time *w* and the linear growth rate *g* are parameters of the fit, and the best fit is obtained by minimizing the root mean square error (RMSE) for each cascade. Note that 5.7% of the cascades are filtered out because they are too small to provide a fit.

**Fig 10:**
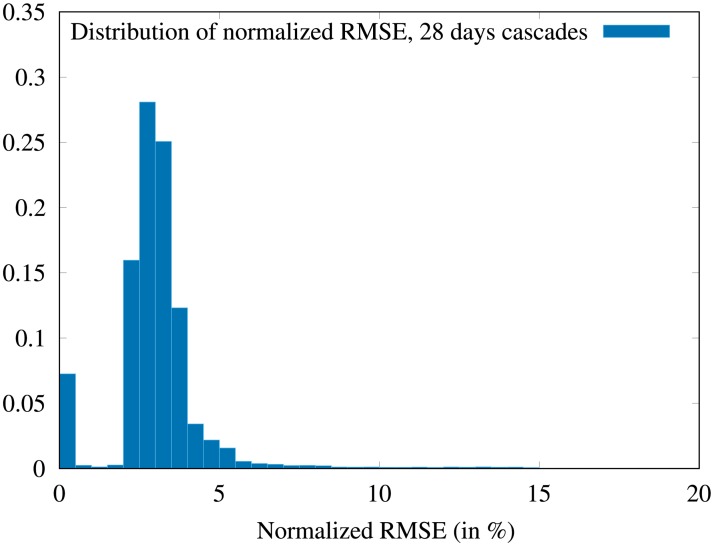
Distribution of the normalized root mean square errors. The RMSE of the fits is computed over the sample of 4-weeks cascades, then it is normalized to the final value of the size of the cascade (*n*_*f*_). 5.7% of the cascades are filtered out as they are too small to provide a fit. We observe that 96.5% of the remaining cascades exhibit a normalized RMSE lower than 0.05.

In order to evaluate the overall quality of the fits, we plot in Fig 10 the distribution of the normalized RMSE (denoted NRMSE) for the 4-weeks cascades (year 2015). We use a standard normalization by *n*_*max*_ − *n*_*min*_ = *n*_*f*_. It can be seen on the distribution that 96.5% of cascade fits have a lower than 0.05 NRMSE. To give the reader an idea of what it visually means, the case represented in Fig 9 has a NRMSE in the interval [0.020; 0.025]. Also, notice that few cascades have a NRMSE lower than 0.02, that is because the model neglect the weekly variations of the infection growth, which entails a small error on most fits, as in the case represented in Fig 9.

**Fig 11:**
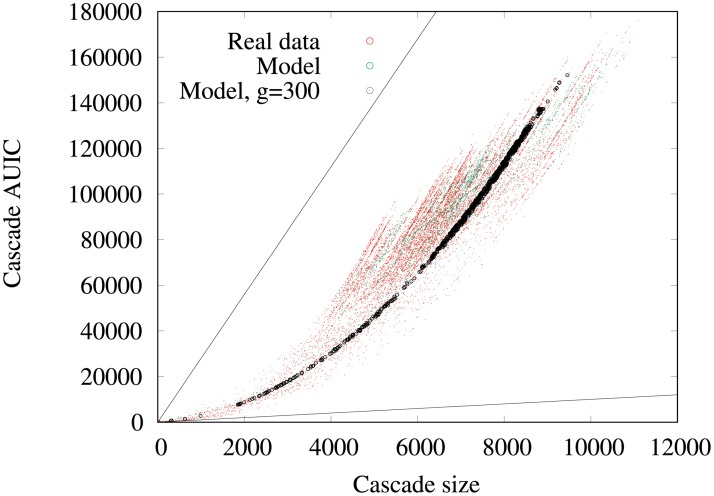
AUIC vs number of infected nodes (4-weeks): Model. Scatter plot of the 4-weeks cascade sizes and *AUIC* in the 2-phase model. The black solid lines represent the theoretical boundaries, black dots correspond to model cascades with a similar linear growth rate (300 ± 5 holdings infected per day).

We present in Fig 11 the plot of the *AUIC* as a function of the final number of infected nodes obtained using this two-phase model (in the 4-weeks cascade case). The model displays the same wing-like envelope as real data does. Furthermore, when setting the value of the growth rate, the points corresponding to the model all fall on a same parabola, which could be inferred from the expression of the *AUIC* as a function of ${\hat{n}}_{f}=\hat{n}\left(t_{0}+\Delta \right)$:

$$\begin{array}{c} {\hat{n}}_{f}=g\cdot \left(\Delta -w\right)+1 \end{array}$$

$$\begin{array}{c} \Rightarrow AUIC=\Delta +\left({\hat{n}}_{f}-1\right)\frac{\left(\Delta -w\right)}{2}=\frac{{\left({\hat{n}}_{f}-1\right)}^{2}}{2g}+\Delta \end{array}$$

This expression reveals indeed that the *AUIC* is a quadratic function of ${\hat{n}}_{f}$, which parameters only depend on *g*. This simple model is then sufficient to understand the salient features of the spreading cascade. Note that the model still fits well the data at a quarterly timescale, but the fit is less convincing on larger timescales.

**Fig 12:**
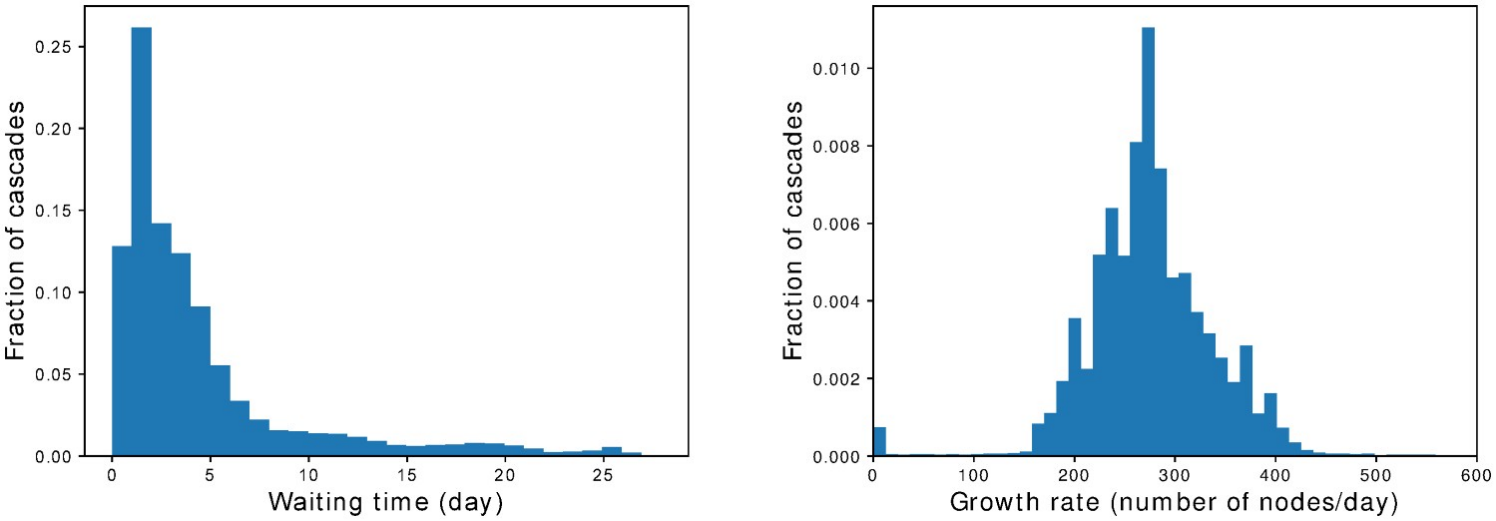
Waiting times and growth rates. Normalized distributions of waiting times (left) and growth rates (right) of 4-weeks cascades, obtained when fitting *n*(*t*) with the two-phase model. While waiting times distribution is heterogeneous, growth rates are distributed homogeneously around approximately 280 infected nodes per day.

We plot in Fig 12 the distributions of waiting times and growth rates according to the model. Waiting times are heterogeneously distributed, with a large majority of cascades having small waiting times. By contrast, growth rates are distributed rather homogeneously: almost 90% of growth rates fall in the range from 150 to 400 infected nodes per day. The distribution is centered around a peak at about 280. Note also that there is a peak at very low growth rates (a few units), which correspond to cascades that do not reach their tipping point.

To conclude, we underline the fact that the growth phase on the BDNI is well fitted by a linear approximation. Moreover, our typical time scales of observation of animal trade networks do not allow to observe the saturation effect. What we observe is a transient. We mean by *saturation effect* that the growth rate of the infection should notably decrease and turn sublinear, because the infections run out of susceptible nodes to infect. The saturation effect begins to be clearly visible on *n*(*t*) for a Δ of several years (not shown in this work).

#### Impact of the type of nodes

**Fig 13:**
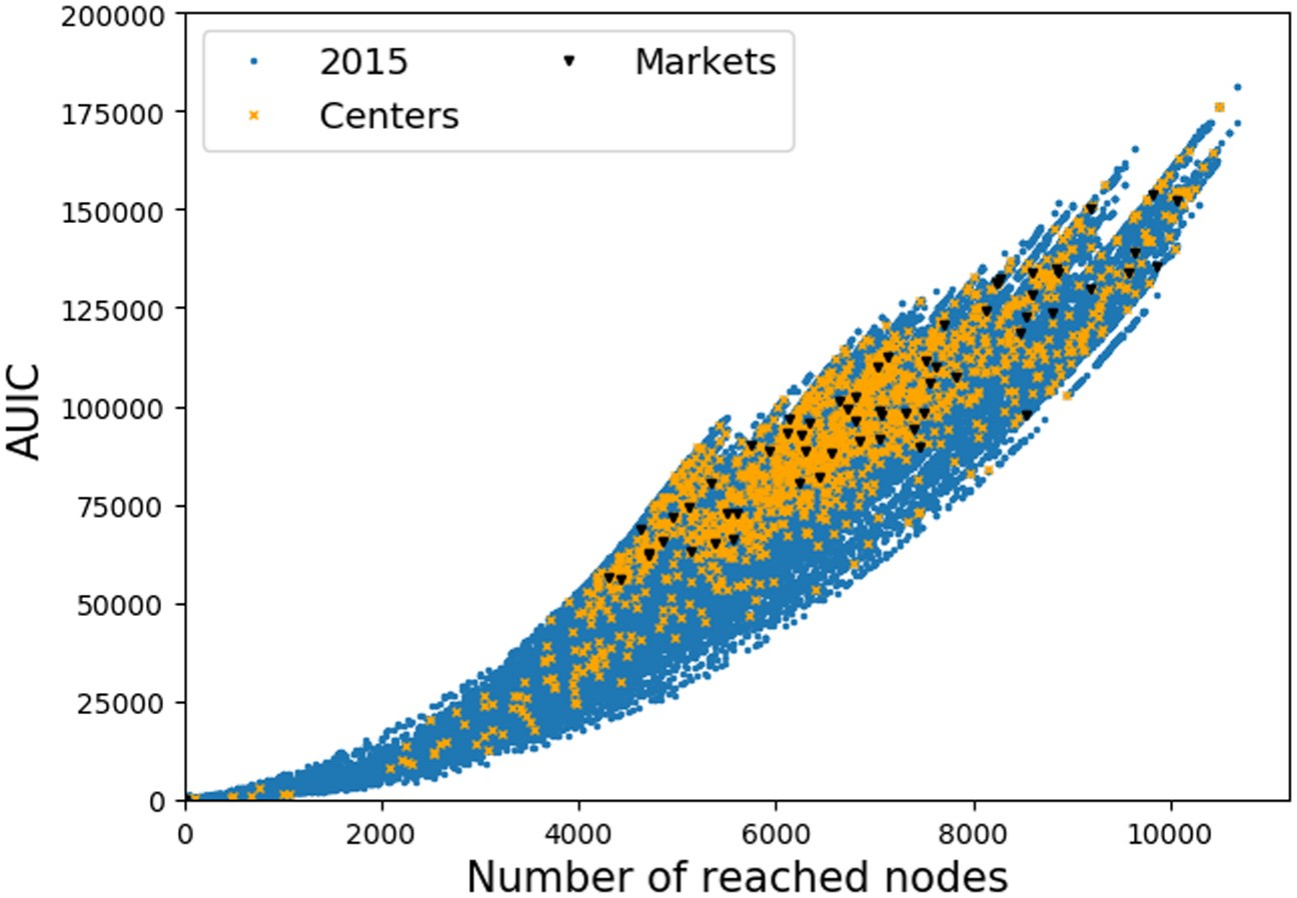
AUIC vs n(t): Role of node types. *AUIC* as a function of the final number of reached nodes (4-weeks cascade on year 2015). Cascades are differentiated according to the type in the BDNI of their source nodes: markets (black), assembly centers (yellow) and farms (blue). The plot indicates that centers and markets tend to be sources of larger cascades with larger *AUIC* than farms.

Before moving to the implications of what we have observed on the epidemic control strategies, let us briefly discuss the role of the node types (farm, assembly center or market) on the scatter plot that we have discussed. Intuitively, waiting times can be interpreted as the times needed to reach some key nodes in the network. We have seen previously that markets and assembly centers are very active and thus likely to be among these key nodes. To test this assumption, we plot in Fig 13 the *AUIC* as a function of *n*_*f*_, depending on the type of the starting node of a cascade.

For a given number of reached nodes, the figure shows that propagations starting from markets have *AUIC* in the upper part of the scatter plot, which means that these cascades are among the fastest on average. Moreover, we notice that cascades starting from markets almost always reach more than 4000 nodes, therefore, markets source nodes lead to fast and large spreadings. Concerning assembly centers, we observe a similar trend, but less dramatic than for markets. In the light of these observations, one can suppose that if a cascade starting from a farm reaches rapidly a market or an assembly center, it is more likely to be fast and large. However, the spreading dynamics is not so simple as to say that an infection reaching a market or an assembly center also reaches its tipping point. This calls for further work to highlight the role of markets and assembly centers in the propagation process.

### Consequences on epidemic control strategies

In this section, we follow a procedure to evaluate epidemic control strategies based on the network structure analysis, which has become popular since early works such as [7]. The overall idea is to identify central elements of the network, in general nodes—sometimes links—and then evaluate how removing them decreases the epidemic risk. The deletion operation represents the fact that sanitary measures are enforced (vaccination, quarantine, slaughter, etc.). We investigate how the elements that we have discussed in the previous sections should be taken into account for this kind of analysis.

#### Elements of the protocol

We consider three key elements underlying this method to assess epidemic control strategies:

- First, the cost of a strategy is usually evaluated using the number of nodes removed from the network.
- Second, the epidemic risk evaluation: according to a static, snapshot-based point of view, it is often made using the connected components of the graph [24, 27, 30, 43]. A natural equivalent in the context of temporal networks are the spreading cascades aforementioned. That’s why recent studies have utilized the outgoing infection chains or related measurements to evaluate the risk in animal trade networks [5, 28, 32, 41, 42].
- Finally, the strategy itself relies on an evaluation of the centrality of a node (or link). A centrality is a measure of how important an element of the structure is in respect to a specific property, here its ability to spread an infection. When the complete structure of the network is available, usual centrality candidates are degree, activity, betweenness, closeness, which are graph-based measurements [5, 7, 27, 30, 32, 41] and more scarcely dynamic centralities [28, 42].

#### Adapting the protocol to temporal networks

We have previously mentioned that a same cascade size could correspond to very different spreading scenarios. Consequently, we have defined the *AUIC* in order to account for the spreading speed. In the following, we use this measurement to assess the epidemic risk. Note that in the case of the BDNI, analyzing the sizes of the cascades or the *AUIC* leads to the same conclusions on target control strategies, certainly because most large cascades follow the same 2-phases scenario that we have described earlier. However, we suggest that with other datasets or other spreading contexts, this measurement should complete an analysis based on outbreak sizes. Indeed, the *AUIC* plays a role which can be compared to the measurement of the epidemic peak in the case of more elaborate epidemic models (such as SIS or SIR).

Another important point is that the atom of information in a link stream is a temporal link (*t*, *i*, *j*). Thus, it makes more sense to evaluate the cost of a strategy using the number of triplets removed rather than the number of nodes or links. Moreover, it makes possible to compare strategies which target different elements of the temporal network structure.

#### Retrospective analysis

We analyze the efficiency of target control strategies in the following way: first we rank nodes, links or triplets according to some centrality measurement. Then, we delete a given fraction of the top-ranked triplets and run the SI spreading model on the remaining structure. We evaluate the efficiency of the strategy by measuring the *AUIC* (and the size, not reported here) of the cascades. This analysis is retrospective in the sense that the data which is used to rank elements of the temporal network structure is also the data on which we run the SI cascades. Experiments are conducted using year 2015 as the observation period and with 4 weeks cascades.

Various strategies are tested in this section:

- We use classic node-based centralities: out-degree (denoted strategy n-OD), out-activity (n-OA) and betweenness centrality (n-BC). Note that computing betweenness centrality is expensive, thus we approximate the computation using the random pivot strategy described in [44], with 20% of nodes used as pivots.
- We also use a basic link-based strategy based on its activity: the number of occurrences of the link over the whole observation period (l-A).
- Finally, we define strategies which are specific to the cascade definition: we compute the number of occurrences of nodes (n-Oc), links (l-Oc) and triplets (t-Oc) in the cascades. The purpose of these strategies is to target nodes, links and triplets which are really located on the propagation paths.

When a strategy is node-based (respectively link-based), removing a node corresponds to removing all triplets, i.e. temporal links, involving this node (resp. link). This has a significant impact on the strategies evaluation. Indeed removing the top 1% (resp. 10%) nodes ranked by decreasing activity corresponds to removing 46% (resp. 80%) of triplets. Consequently, removing even a few central nodes from the cattle trade network has a dramatic impact on the volume of batches exchanged, which suggests that the corresponding safety measures are hardly applicable.

**Fig 14:**
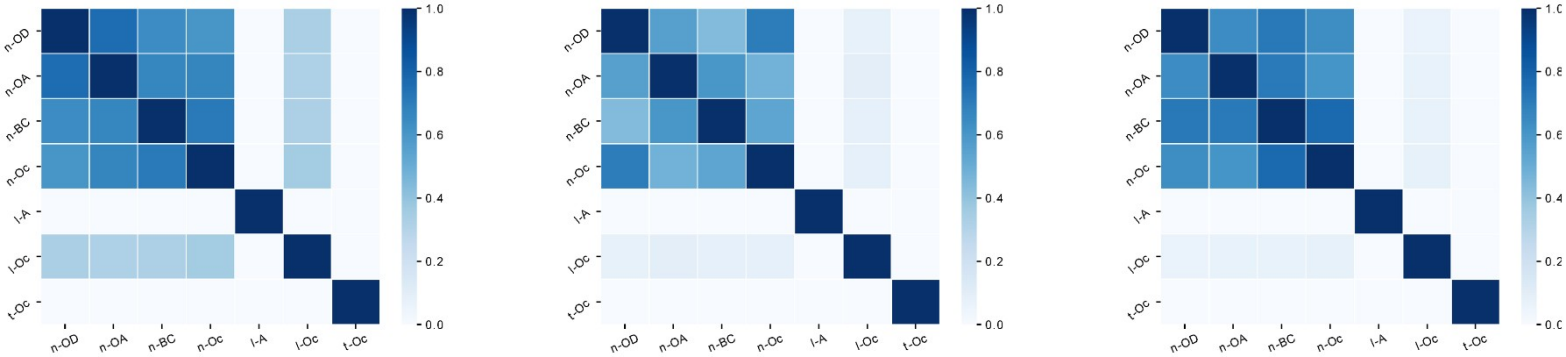
Correlation between strategies. Matrix of the Jaccard index between the top-70,000, top-200,000 and top-400,000 elements in the rankings. The matrices show that the level of correlations between node-based strategies is high, which means that there is an important overlap between the sets of triplets ranked high by two of these strategies.

To get insights about how similar these rankings are, we build sets of 70,000, 200,000 and 400,000 elements (that is approximately a fraction of respectively 2.6, 7.5 and 15% of the triplets that year) featuring the top of each rankings, then we compute the Jaccard index between these sets. Let us remind that the Jaccard index of two sets *A* and *B* is $\frac{\left|A\cap B\right|}{\left|A\cup B\right|}$. The results are reported in matrices displayed in Fig 14. The most striking observation is that all node-based strategies have a relatively high Jaccard coefficient (larger than 0.5), which means that they share more than 2/3 of their triplets. Other rankings are more dissimilar, in particular strategies based on the temporal description.

**Fig 15:**
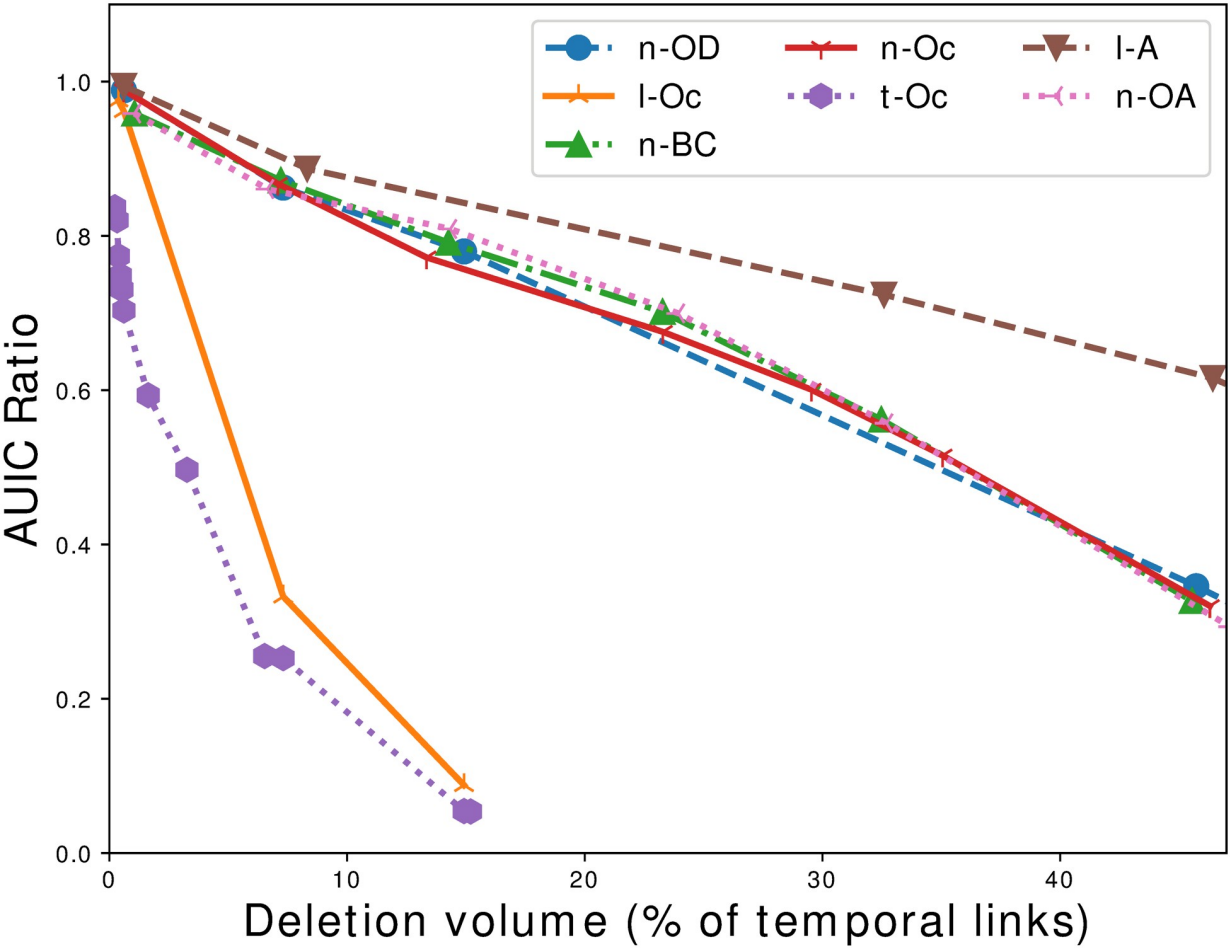
Comparing strategies—Case 1. Comparison of deletion strategies in a retrospective analysis context expressed with the *AUIC* ratio, i.e. the fraction of the maximum *AUIC* to its initial value, as a function of the percentage of triplets deleted. Triplets are ranked according to measures realized on the data of year 2015, and *AUIC* ratios are also measured using cascades of year 2015. The plot shows that strategies based on the number of occurrences of links (l-Oc) and the number of occurrences of triplets in the cascades (t-Oc) are much more efficient than all other strategies, in particular node-based strategies (n-OD, n-OA, n-BC, n-Oc).

Going back to the comparison of target control strategies, the results are gathered in Fig 15. We observe that the least efficient strategy is the one based on the number of occurrences of a link on the observation period (l-A). For instance, when removing approximately 200,000 temporal links (7.5% of the total), the maximum cascade *AUIC* drops by 6% only. All three node-based strategies are a little more efficient, leading to a 9% drop for a 7.5% triplet deletion. Contrary to what has been reported in other works [24, 30], betweenness centrality does not outperform other node-based methods significantly. A reader may be surprised by these observations, but it stems from the fact that we are actually examining very low percentages of node deletions (7.5% of triplets deleted corresponds to only around 10 nodes deleted in node-based strategies). The fact that node-based methods are almost identically efficient is consistent with the level of similarity of the rankings that we have mentioned before. By contrast, the strategy based on the number of occurrences of a triplet in a cascade and the number of occurrences of a link in a cascade are much more efficient. For example, they lead to respectively a 77% and 67% drop with 7.5% triplet deleted. Furthermore, these strategies allow to rank only around 400,000 triplets, beyond that links simply do not appear in any cascade of the sample. We could find the remaining triplets by extending the sample size, but the interest would be limited as we have nearly completely contained any outbreak. In [29], the authors stated that only a small fraction of nodes could lead to large cascades, if they are reached at the right time. In the light of these observations, we can go one step further by stating that only a fraction of the links could lead to large cascades.

As a conclusion, the number of occurrences of links and triplets in the cascades are the most efficient targeted control methods on the temporal networks that we have tested. One could argue that it comes from the fact that we are using a measure which is related to the structure of the cascade, that is to say what is used to evaluate the risk. Still, the cascades used for ranking are different from the cascades used to evaluate the performance (in particular they don’t have the same starting point *t*_0_).

#### Predicting future risks

Now in a real prediction context, a forecaster would have to use past data in order to evaluate the effect on cascades in the future. In order to be efficient, a prediction protocol necessitates that the structure of the network does not evolve too fast, as discussed in previous studies [27, 30, 45]. In this section, we evaluate if the strategies identified in the previous section can be used for risk prediction.

So we apply the same experimental protocol, except for the fact that we use data from the previous year (in our experiments, 2014) to rank nodes, links or triplets; and evaluate the strategies by running the SI model on the following year (2015). Note that with this setting, some of the strategies defined previously cannot be used: in particular, as triplets from past year obviously differ from triplets of the current year, we cannot rank triplets of the current year following the strategy based on the number of occurrences of triplets in cascades of the previous year.

**Fig 16:**
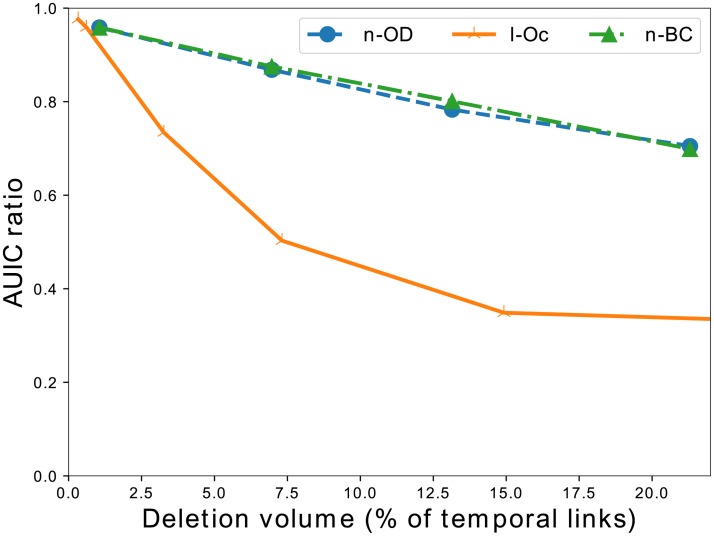
Comparing strategies—Case 2. Comparison of deletion strategies in a predictive context expressed with the *AUIC* ratio, i.e. the fraction of the maximum *AUIC* to its initial value, as a function of the percentage of triplets deleted. Triplets are ranked according to measures realized on the data of year 2014, and *AUIC* ratios are measured using cascades of year 2015. The plot shows that the strategy based on the number of occurrences of links (l-Oc) in the cascades is much more efficient than other strategies, in particular node-based strategies (n-OD, n-BC).

We report on Fig 16 the corresponding results. Here again, node-based methods perform more or less similarly, but the method based on removing links that occurred frequently in the past cascades is much more efficient. However, we observe that after a number of deletions (again around 400,000 deletions, or 15% of the triplets), the strategy does not improve anymore, as we probably cannot find the remaining triplets involved in the cascades. The remaining cascades cannot be broken probably because they involve links or nodes that did not exist during the previous year.

When measuring what types of links are removed depending on the types of nodes that they connect, we observe that the links from centers to farms are heavily overrepresented among the top links. For example they represent 80% of the links deleted among the first 200,000 triplets, while they represent only 13% of the links in the data. This share decreases when deleting more triplets, mostly to the benefit of links connecting farms to centers which are underrepresented in the top of the ranking. We interpret these observations as the existence of some kind of essential links in the network, which are regularly used for cattle trade and are also bottlenecks of the overall flows of animals over the network. Links from centers to farms seem to be overrepresented among them. These links are priority targets for control strategies, as deleting them during a period of time should be less costly than deleting a node from the network. It is tempting to assert that such links constitute a backbone, but previous works on the topic have emphasized the fact that a stable backbone is hardly observable in these networks [27, 45]. While this is true that the renewal rate of the links is high in cattle trade networks, these recurring links play an important role in the spreading process which justifies to focus on them.

#### Effect on the cascade growth rates

**Fig 17:**
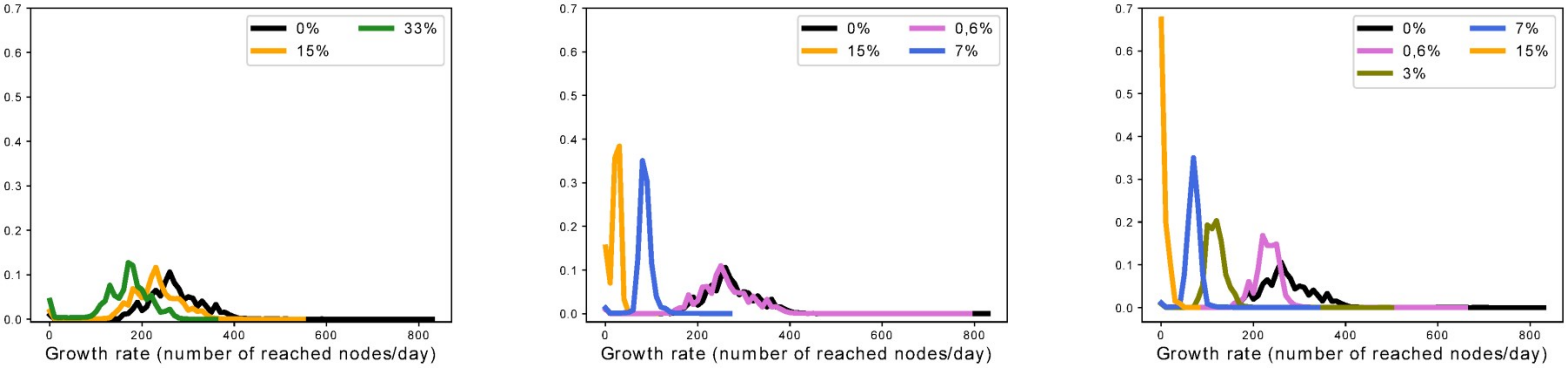
Effect on the distribution of growth rates of deletion strategies. Effect of suppressing triplets according to different strategies on the distribution of growth rates computed with the two phases model. Left: node out-degree strategy, middle: occurrences of links in a cascade strategy, right: occurrences of triplets in a cascade strategy. While node-based deletion strategies imply lower averages, they do not change the shape of the growth rates distributions; in comparison link and triplet-based strategies deeply disrupt the distributions.

We come back to the two-phase model presented previously to analyze the node deletion processes. When deleting triplets according to the retrospective analysis protocol, we observe that the two-phase model still fits the observations relatively well with much smaller cascades. Moreover, when fitting the growth rates, we observe that different strategies affect the distributions very differently—see Fig 17. Indeed, in the case of node-based deletion strategies, we can see that the average diminishes but the shape of the distribution remains qualitatively similar. By contrast, deletion strategies based on the occurrence of links and triplets in a cascade not only cause the average to be lower, but also distort the distributions: cascades tend to have homogeneous growth rates. We analyze this observation in the following way: using node-based strategies, a node deletion cuts out a branch of the cascade but does not modify its shape significantly, most of the nodes that could be reached are still infected using secondary paths; whereas the link and triplet-based strategies deeply alter the shape of the cascade by cutting selectively bottleneck paths.

## Discussion

In this work, we gave support to the idea that it is crucial to take into account the temporal dimension of data in order to model spreadings in animal trade networks. This point of view is well-spread in the literature, and previous works put forward several arguments in its favor. In the case of the BDNI, we confirmed several aspects of these works, in particular the fact that snapshot-based representations tend to overestimate the sizes of outbreaks, and give a distorted view of the distribution of potential outbreak sizes. Furthermore, we investigated the spreading scenarios encountered, using not only measurements of the outbreak size but also of its speed. We pointed out that our spreading process leads essentially to a unique kind of large-size cascades in the case of the BDNI, well approximated by a two phases model. This model gives a simple picture of the spreading process: during a waiting period, the infection remains nearly silent, then it reaches a tipping point and grows linearly from then on. These observations led us to reconsider several aspects of epidemic control strategies. First, the cost of a strategy should be more appropriately evaluated in terms of the natural unit of a temporal network, that is a triplet of interaction. Second, the impact of an infection model may be assessed with its size but also with its speed, even in the case of our standard deterministic SI model. Finally the strategy itself can be conceived using the epidemic cascades themselves, which proves to be much more efficient that usual node-centrality based strategies, in a retrospective as well as in a predictive context.

This study raises several questions, most notably the identification of essential links in a directed temporal network, which would be favored routes for spreading phenomena. Links belonging to many cascades are certainly among those links, and we observed in the BDNI that links from centers to farms are largely overrepresented in this set. We leave for future work a more comprehensive investigation of the patterns formed by these essential links: it would be indeed very interesting to point out more elaborate temporal motifs which are often part of spreading cascades.

From our point of view, another interesting point is that we did not detect the saturation effect of the spreading phenomenon at the timescales of observation. There are two possibilities: either the saturation effect is occurring on a larger timescale, or there are new holdings entering the system which always feed the cascades with new nodes. In the second case, we would never observe the saturation phenomenon, and if the renewal rate is high enough, the linear growth could continue forever. The truth lies in between these two extreme cases. A few additional experiments indicate that it is closer to the former hypothesis: indeed, after a few years the renewal rate per day seems to stabilize around a few dozens of new nodes appearing in the dataset per day, which is insufficient to account for the typical growth rate of the cascades. However, a question to solve for future works is to determine what part of the growth rate is explained by new nodes entering the system and what part comes from old nodes which have not been reached yet.
